# Combinatorial Strategies to Target Molecular and Signaling Pathways to Disarm Cancer Stem Cells

**DOI:** 10.3389/fonc.2021.689131

**Published:** 2021-07-26

**Authors:** Giuliana Catara, Antonino Colanzi, Daniela Spano

**Affiliations:** Institute of Biochemistry and Cell Biology, National Research Council, Naples, Italy

**Keywords:** cancer stem cells, cancer, combinatorial strategies, signaling pathways, molecular pathways

## Abstract

Cancer is an urgent public health issue with a very huge number of cases all over the world expected to increase by 2040. Despite improved diagnosis and therapeutic protocols, it remains the main leading cause of death in the world. Cancer stem cells (CSCs) constitute a tumor subpopulation defined by ability to self-renewal and to generate the heterogeneous and differentiated cell lineages that form the tumor bulk. These cells represent a major concern in cancer treatment due to resistance to conventional protocols of radiotherapy, chemotherapy and molecular targeted therapy. In fact, although partial or complete tumor regression can be achieved in patients, these responses are often followed by cancer relapse due to the expansion of CSCs population. The aberrant activation of developmental and oncogenic signaling pathways plays a relevant role in promoting CSCs therapy resistance. Although several targeted approaches relying on monotherapy have been developed to affect these pathways, they have shown limited efficacy. Therefore, an urgent need to design alternative combinatorial strategies to replace conventional regimens exists. This review summarizes the preclinical studies which provide a proof of concept of therapeutic efficacy of combinatorial approaches targeting the CSCs.

## Introduction

Cancer stem cells (CSCs) constitute a cell subpopulation within the tumor whose frequency depends on the tumor type (1) and stage, being the frequency increasing with the tumor malignant progression (2). These cells possess both the ability to unlimited self-renewal and to generate the heterogeneous and differentiated cell lineages that form the tumor bulk (3). In addition, they show enhanced ability to form tumorspheres (4), high tumorigenicity (5) and high metastatic potential (6). CSCs have a leading role on resistance to cancer therapies such as chemotherapy, radiotherapy and molecular targeted therapy (7, 8). In fact, recent findings show that these conventional therapeutic interventions exert selective pressure on tumors (9, 10) resulting in the activation of or selection of cancer cells unresponsive to the treatment that display alternative pathways associated with CSCs properties. High expression level of transmembrane proteins adenosine triphosphate-binding cassette family which efflux anti-tumor drugs out of tumor cells (11), elevated capacity of DNA repair mechanism, increased protection against reactive oxygen species (ROS) and up-regulation of anti-apoptotic pathway (12) constitute a range of CSCs properties that serve chemotherapy and radiotherapy resistance. In addition to the above-mentioned features, CSCs acquire a transient state of slow proliferation that identifies a population of quiescent cells able to maintain viability in conditions that kill the other cancer cells. However, after the discontinuation of the therapy, the quiescent state is reverted and CSCs can regenerate cancer (13) following the activation of new survival strategies, including new mutations, trans-differentiation or reprogramming (14). Moreover, the radiotherapy induces the Epithelial to Mesenchymal Transition (EMT) program that makes the CSCs more invasive and resistant to the therapy (15, 16).

Developmental signaling pathways [including Wnt, Sonic Hedgehog (Shh) and Notch] and oncogenic cascades (including transforming growth factor-beta (TGF-β), Janus kinase/signal transducer and activator of transcription 3 (JAK/STAT3), phosphatidylinositol 3-kinase/V-Akt murine thymoma viral oncogene/mammalian target of rapamycin (PI3K/AKT/mTOR), Mitogen-activated protein kinase (MAPK) and V-SRC avian sarcoma (Src) have been identified as key players both in CSCs biology maintenance and in cancer therapy resistance (17, 18). The description of these signaling pathways and their roles in defining the biology of CSCs will not be tackled herein and the reader is invited to refer to reviews that widely discuss these topics (17–28). Although several targeted approaches relying on monotherapy have been developed to affect these pathways, they have shown limited efficacy due to the activation of bypass pathway(s) and to the complexity of signaling networks containing feedback loops and cross-talk. For example, bypass activation of mitogen-activated protein kinase kinase (MEK) and/or AKT limits mTOR/PI3K inhibitor therapy (29, 30). Src inhibition rapidly mediates MEK/MAPK induction in preclinical breast cancer models (31, 32) and in high-grade serous ovarian cancer cellular model (33). MEK inhibition induces PI3K pathway in KRAS mutant cancers leading to MEK inhibitor resistance (34). The activation of MAPK-interacting kinase (MNK) signaling in response to mTOR complex1 (mTORC1) inhibition leads to the resistance mechanism in medulloblastoma (35). Therefore, strategies targeting more than one pathway might yield greater, and more durable responses. Similarly, strategies that combine the molecular targeted therapy with chemotherapy or radiotherapy could be more effective. In addition to signaling pathways, tumor-associated antigens, or molecules expressed by CSCs, have been linked to therapeutic resistance. These molecules represent targets for the development of new therapeutic avenues.

**Figure 1:**
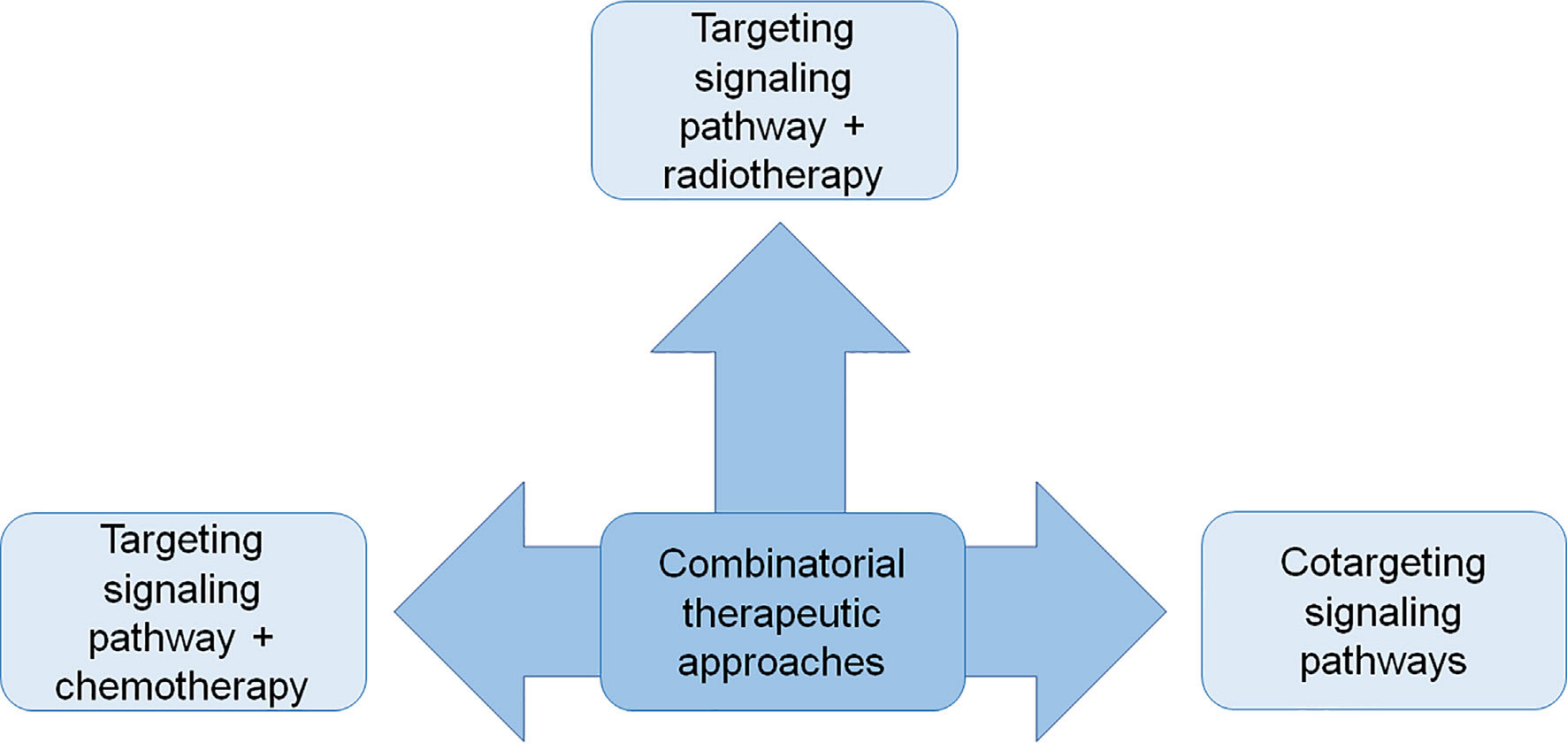
The schematic diagram shows combinatorial strategies to fight CSCs applied in the preclinical studies described in the text.

This review describes the state of the art of the CSCs-targeted combinatorial therapies investigated in the preclinical studies with a focus on the hematological malignancies and different types of solid tumors. These studies provide a proof of concept of the efficacy of these dual therapeutic approaches, in which the molecular targeted therapy, and/or the chemotherapy and/or the radiotherapy are combined each with other, to fight CSCs (**Figure 1**).

## Combinatorial Strategies in Treatment of Hematological Malignancies CSCs

Combinatorial therapeutic approaches have been investigated in treatment of acute myeloid leukemia (AML) and chronic myeloid leukemia (CML) CSCs.

### Acute Myeloid Leukemia

About 30% of AML patients show *FMS-like tyrosine kinase-3* (*FLT3*)-mutations, which are associated with high levels of β-catenin. This observation suggests that the combined blockade of β-catenin and FLT3 could represent a new therapeutic avenue. Here AML and Leukemia Stem Cell (LSC)/progenitor cell viability is inhibited following treatment with the β-catenin/CBP antagonist C-82 combined with FLT3-tyrosine kinases inhibitor (TKI) sorafenib or quizartinib (36). Pre-treatment with C-82 sensitizes cells to sorafenib or quizartinib, thus resulting in reduced cell viability and induction of apoptosis both in AML cells and LSC/progenitor cells. Similarly, *in vivo* C-82 in combination with sorafenib or quizartinib synergistically acts to promote prolonged survival in mice xenografted with either a *FLT3*-mutated cell line or patient-derived tumor cells (patient-derived xenograft (PDX) mice). Mechanistically, C-82, TKIs or the combination of the two drugs effectively inhibit Wnt/β-catenin targets and FLT3 downstream signaling, with the combinatorial administration being more effective (36). The efficacy of the therapy has been also tested by using PRI724, another β-catenin/CBP antagonist. In addition, the cooperative action supported by blockade of Wnt/β-catenin and FLT3 is also provided by the administration of dual FLT3 and Wnt/β-catenin inhibitor SKLB-677 that suppresses LSCs and has strong anti-leukemia activity in *FLT3*-mutated AML cells (37), thus providing the rationale for the development of clinical trials.

Shh activation is detected in 45% of AML biopsies (38) and plays a role in drug resistance of AML CSCs, thus providing the rationale for selective inhibition of this pathway for AML treatment. Cyclopamine is a Shh inhibitor that does not affect the survival of normal hematopoietic stem cells (39, 40). A therapeutic combinatorial strategy relying on the administration of cyclopamine in the presence of either Lipopolysaccharide (LPS) or Tumor Necrosis Factor-α (TNF-α) or interferons (IFNs) has been proposed to cure AML patients (41). Here cyclopamine-LPS administration synergistically reduces cell viability respect to a moderate decrease for cells treated with cyclopamine alone or LPS alone and leads to a massive cell apoptosis in THP-1 and U937 cells, AML patients cell lines and AML primary cells. In *in vivo* AML xenograft mouse model, in which U937 cells were injected in severe combined immunodeficient (SCID) mice, combinatorial therapy determines a reduction in tumor growth. Similar results are obtained replacing LPS with TNF-α or IFNs, which allows to reduce the side effects caused by LPS. To exclude an eventual off-target effect another Shh pathway inhibitor, SANT-1, was administered in combination producing same effects. Thus, this synergistic suppressive activity of Shh inhibitors in combination with either LPS or TNF-α or IFNs may be exploited to induce cell death in other types of cancer cells.

Aiming at overcoming resistance of LSCs to chemotherapy in patients with AML, an additional combined therapy targeting Shh signaling components has been proposed by Long and collaborators (42). The study provides evidence that Gli1 could be a promising target for AML therapy. As such, Gli1 is highly abundant in AML CD34^+^ LSCs and in samples derived from AML patients in comparison to the healthy donors. Targeting Gli1 by the small-molecule inhibitor GANT61 significantly inhibits AML cells growth, suggesting that Gli1 can be considered a promising target to cure AML patients. Of note, co-treatment with Gli1 inhibitor and the generally used anti-leukemic drugs, such as cytarabine or all trans retinoic acid (ATRA), synergistically reduces AML cells viability, showing that pre-treatment with GANT61 promotes sensitization of primary AML CD34^+^ LSCs to anti-leukemic drugs. These data foster the findings obtained in previous studies where the combination of GANT61 with vincristine reverses chemoresistance in Lucena-1 myeloid leukemia cells (43).

Raf/MEK/ERK (MAPK) cascade is activated in 70%-80% of patients affected by AML and regulates Bcl-2 family proteins by stabilizing anti-apoptotic Mcl-1 and inactivating pro-apoptotic BIM (44, 45). Moreover, MAPK signaling activation contributes to acquired resistance to venetoclax, a strong inhibitor of Bcl-2, which is highly expressed in AML LSCs. Starting from these findings, anti-leukemia effects of concomitant Bcl-2 and MAPK blockade by venetoclax and MEK1/2 inhibitor cobimetinib in AML were investigated (46). The combined treatment suppresses both Bcl-2:BIM and Mcl-1:BIM complexes, enabling release of free BIM to induce apoptosis in AML stem/progenitor population. In addition, the combination significantly suppresses the clonogenic potential of myeloid progenitors compared to either drug alone and importantly minimally affects normal progenitor function (46).

Dasatinib is a highly potent inhibitor of tyrosine kinases, including Src and ABL, approved for treatment of solid tumors and hematological malignancies (47, 48). It was used in combination with natural compounds or chemotherapeutic drugs for CSCs treatment. Dasatinib was found able to enhance both *in vitro* and *in vivo* the sensitivity of AML stem/progenitor cells to chemotherapeutic agents such as daunorubicin, cytarabine and doxorubicin (49). Here the *in vitro* combined treatment results in a significant increase in inhibition of AML stem/progenitor cell proliferation and colony-forming cells compared to single agent treatments. Moreover, the dual treatment significantly enhances the apoptosis of AML stem/progenitor cells (49). In *in vivo* AML mouse model, dasatinib and chemotherapy (cytarabine or doxorubicin) combination shows significantly prolonged survival, indicating improved targeting of AML LSCs (49). On a molecular point of view, in AML stem/progenitors cells, the combination inhibits the activation of AKT and MAPK signaling pathways, while does not affect STAT5 pathway, and enhances the expression levels of p53 and its target genes, including BAX, p53-upregulated modulator of apoptosis (PUMA), p21, NOXA and DR5. In turn the reduced activation of AKT causes a reduction of HDM2 serine 166 phosphorylation, which is associated with p53 degradation. Therefore, the inhibition of AKT pathway probably represents the molecular mechanism responsible for p53 increased level, which is functional for the elimination of AML stem/progenitor cells treated with dasatinib/chemotherapy combination (49).

The relevance of blockade of PI3K/AKT/mTOR signaling cascade in AML stem cells therapy is highlighted by the combined treatment of active-site mTOR inhibitor MLN0128 and HDAC3 inhibitor vorinostat which stimulates a significantly higher apoptosis induction than the single agents in AML stem cells (50).

### Chronic Myeloid Leukemia

CML is a stem cell disease characterized by the reciprocal translocation t(9;22) that generates the BCR-ABL1 tyrosine kinase oncoprotein. The therapy consists in the administration of BCR-ABL1 TKI, however the TKI resistance occurs in a large number of patients. The molecular mechanisms responsible for TKI resistance consist in the generation of BCR-ABL1 mutations, intrinsic stem cell resistance (51) or, within the native BCR-ABL1 genetic background, in the activation of pro-oncogenic signaling networks and molecules, including AKT, mTOR, MEK, STAT3, STAT5, JAK2, or Src kinases (52–56). In particular, the activation of STAT3 signaling pathway protects CML cells upon TKI-mediated BCR-ABL1 inhibition (54) and confers kinase-independent TKI resistance to primary CML stem and progenitor cells (57). The STAT3 targeting with BP-5-087, a potent and selective STAT3 inhibitor, in combination with TKI imatinib rescues the TKI sensitivity of TKI-resistant CML cell lines and primary CML stem and progenitor cells with no toxicity to normal hematopoietic stem or progenitor cells (57). Therefore, the combined blockade of both BCR-ABL1 and STAT3 significantly decreases the survival and the clonogenic properties of CML stem and progenitor cells with kinase-independent TKI-resistance compared to inhibition of only BCR-ABL1 or only STAT3, thus suggesting that the combination could be a new therapeutic approach for eradicating the TKI-resistant stem cell population in CML affected patients. Based on these results, another combined therapeutic approach that uses the STAT3 potent inhibitor CDDO-Me was assessed in CML treatment. The combination of CDDO-Me and TKI ponatinib induces the apoptosis and reduces the clonogenic ability of both CML stem cells and progenitor cells in a synergistic manner, as a result of the full and simultaneous inhibition of both STAT3 and STAT5 (58). CDDO-Me shows pleiotropic effects; in fact, it, in addition to inhibit STAT3, induces ROS generation, suppresses several survival-related molecules (including AKT, mTOR, and MAPK), and increases the expression of heme-oxygenase-1, a heat-shock-protein that contributes to drug resistance of CML cells (59). For these reasons, the combined inhibition of STAT3, heme-oxygenase-1 and BCR-ABL1, by using CDDO-Me, the heme-oxygenase-1 inhibitor SMA-ZnPP and TKI (dasatinib or ponatinib) respectively, was also investigated. The anti-proliferative effect of the triple-combination in CML cells was superior to single drug treatment or 2-drug treatment (58). Interestingly the CDDO-Me/TKI and CDDO-Me/SMA-ZnPP/TKI combinations do not significantly affect the proliferation of normal bone marrow cells, thus further supporting them as new therapeutic avenues (58).

A summary of the preclinical studies performed on CSCs of hematological tumors is provided in **Table 1**.

**Table 1:** Combinatorial therapies tested in pre-clinical studies to treat CSCs from acute myeloid leukemia (AML) and chronic myeloid leukemia (CML).

Tumor	Combinatorial therapy	Molecular target	Phenotypic outcome	Cellular/animal models	References
Acute myeloid leukemia	C-82/PRI724 + sorafenib/quizartinib	CBP/β-catenin + Tyrosine Kinase FLT3	Suppression of CSCs proliferation	AML cells, LSC/progenitor cells	(36)
			Prolonged survival	Mice xenografted with either a *FLT3*-mutated cell line or PDX cells	
Acute myeloid leukemia	Cyclopamine + LPS/TFN-a/IFNs	Smo	Reduction of CSCs viability	THP-1,U937 cells, AML patients cell lines and AML primary cells xenograft mouse model	(41)
			Suppression of tumor growth		
Acute myeloid leukemia	GANT-61 + cytarabine (Ara-c)/ATRA	Gli1	Reduction of CSCs viability	AML cells	(42)
Acute myeloid leukemia	Cobimetinib + venetoclax	MEK1/2 + Bcl-2	Induction of apoptosis and inhibition of progenitor clonogenic properties	AML stem/progenitor cells	(46)
Acute myeloid leukemia	Dasatinib + daunorubicin/cytarabine/doxorubicin	Src	Inhibition of cell proliferation and colony-forming cells, induction of apoptosis	AML stem/progenitor cells	(49)
			Inhibition of AML stem/progenitor cell proliferation and increase of mice survival	AML mouse model	
Chronic myeloid leukemia	BP-5-087 + imatinib	STAT3 + BCR-ABL1	Decrease of survival and clonogenic properties	CML stem and progenitor cells	(57)
Chronic myeloid leukemia	CDDO-Me + ponatinib	STAT3 + STAT5 + BCR-ABL1	Induction of apoptosis and reduction of clonogenic properties	CML stem cells and progenitor cells	(58)
Chronic myeloid leukemia	CDDO-Me + SMA-ZnPP + dasatinib/ponatinib	STAT3 + heme-oxygenase-1 + BCR-ABL1	Inhibition of CML cell proliferation	CML cells	(59)

## Combinatorial Strategies in Treatment of Solid Tumors CSCs

Combinatorial therapeutic approaches have been investigated in treatment of CSCs from solid tumors including central and sympathetic nervous system tumors, breast cancer, prostate cancer, non-small-cell lung cancer (NSCLC), head and neck squamous cell carcinoma (HNSCC), colorectal cancer, hepatocellular carcinoma (HCC), ovarian cancer, pancreatic cancer and melanoma.

### Tumors of Central and Sympathetic Nervous System

#### Gliomas

Gliomas, the most prevalent primary tumors of the brain and spinal cord, are characterized by the amplification of Gli1, a component of the Shh cascade, following the Ras/NFkB-mediated activation of Shh pathway (60). Therefore, the combinatorial targeting of Shh and Ras/NFkB pathways could represent a new strategy to fight gliomas. Driven by this hypothesis, Dixit and collaborators demonstrated that the combinatorial approach using Shh inhibitor SANT-1 and Ras/NFkB inhibitor guggulsterone is effective in arresting glioma CSCs and non-stem cells proliferation (61). The combined treatment significantly increases glioma CSCs and non-stem cells apoptosis compared to monotherapies, thus resulting in decreased cell viability. On a mechanistic point of view, guggulsterone inhibits Ras and NFkB activities and sensitizes cells to SANT-1-induced apoptosis. To corroborate this finding, the inhibition of Ras or NFkB is sufficient to sensitize glioma CSCs and non-stem cells to SANT-1. Of note, treatment with a combination of SANT-1 and guggulsterone on SVG p12 astroglia cell line does not decrease cell viability, thus suggesting that this combination selectively targets the viability of glioma CSCs and non-stem cells without affecting normal astrocytes (61).

Among the gliomas, glioblastoma (GBM) represents the most common and aggressive tumor of the central nervous system. The current standard of GBM treatment is surgical resection, concurrent and adjuvant chemotherapy with temozolomide (TMZ) and radiotherapy (62). However, the glioma stem cells (GSCs), responsible for GBM malignant growth, therapeutic resistance and recurrence, are not sensitive to TMZ and until now, there are no effective drugs for the clinical treatment of these cells. To develop new therapeutic approaches to fight GBM, the therapeutic efficacy of TMZ and demethoxycurcumin (DMC) combination was investigated on GSCs (63). The DMC-TMZ treatment causes a strong impairment of GSCs proliferation due to G0/G1 cell cycle arrest and apoptosis induction. On a molecular point of view, DMC-TMZ combination increases ROS production in GSCs, that in turn promotes the activation of caspase-3 signaling, thus inducing the apoptosis. In addition, the two drugs co-administration leads to a strong reduction of STAT3 signaling pathway activation in GSCs, thus resulting in the inhibition of STAT3 targets genes, including *c-Myc* and *CDC25A* genes, implicated in activating G1 to S cell cycle progression, and *Bcl-2* and *Bcl-xL* genes that promote cell survival (63). In summary, STAT3 signaling blockade caused by DMC-TMZ combinatorial treatment contributes to GSCs growth inhibition and apoptosis, thus representing a new avenue to fight GBM.

GBM shows hyperactivation of Notch signaling, therefore several Notch-targeted therapies based on the γ-secretase inhibitors (GSIs) exploitation have been evaluated either as monotherapy or in combinatorial approaches with other regimens. Currently the GSI RO4929097 has been found effective in the treatment of patients with severe glioma in association with bevacizumab (64). Alternatively, it has been described to act synergistically with chemotherapeutic drugs contributing to reduce the numbers of GBM CSCs (65). One of the most promising approach relies on the combined treatment with GSIs and farnesyltransferase inhibitors (FTIs), the latter evaluated for their potential to cure glioma-affected patients, though with modest outcomes (66, 67). Ma and colleagues have shown that FTIs and GSIs act synergistically on GBM stem cells that become more sensitive to radiation respect to non-stem tumor cells (68). In particular, combined administration of the FTI tipifarnib and the GSI RO4929097 produces anti-proliferative effects and cytotoxicity on CD133^+^ CSCs respect to single agent treatment. On the contrary, CD133^-^ cells were resistant to these drugs used either in mono- or combined therapy. Similarly, GBM xenograft tumors treated with the combination show a reduced tumor growth. The dual therapy tested on orthotopic GBM models extends the mice median survival with two cases of durable response. From mechanistic point of view, the synergistic activity of the cure relies on the ability of FTIs to compromise cell-cycle progression prior of the effect exerted by RO4929097, though the molecular mechanism remains not fully elucidated. On the same line of evidence, valuable outcomes have been reached in other trials using FTIs and GSIs in combination with TMZ (69) or treating other cancers types, such as breast and lung cancers (70, 71).

Another combined therapeutic approach uses LY2109761, an inhibitor of TGF-β type I serine/threonine kinase receptor (TGFβR-I), that shows anti-tumor activity in various tumor models (72–74). The combinatorial effects of radiation and LY2109761 were investigated in *in vitro* established human GBM cell lines and GSCs and in *in vivo* subcutaneous and orthotopic tumor models (75). Here the combined treatment significantly enhances the radiation-induced double-strand breaks, inhibits the DNA repair and increases the apoptosis of both GBM and GSCs. Moreover, it reduces the self-renewal and proliferation of GSCs compared to LY2109761 or radiation monotherapy. These data demonstrate that LY2109761 sensitizes both GBM and GSCs toward radiation (75). On a molecular point of view, combined treatment reduces SMAD2 phosphorylation, thus resulting in the blockade of TGF-β signaling cascade, that in turn affects the expression profiles of genes involved in several functional categories including cellular movement, cell death, and cellular growth and proliferation (75). Furthermore, the combinatorial treatment delays the tumor growth in both GBM subcutaneous and GSCs orthotopic xenograft tumor models and increases the survival of mice bearing orthotopically implanted brain tumor compared to single treatments. In addition, histologic analyses show that LY2109761 inhibits tumor invasion promoted by radiation, reduces tumor microvessel density, and attenuates mesenchymal transition (75). These findings demonstrate the therapeutic efficacy of this approach in *in vitro* and *in vivo* preclinical models of GBM.

Evidence demonstrates that the cross-talk between MEK/ERK and PI3K/mTOR pathways controls the maintenance of self-renewal and tumorigenicity of GBM CSCs (76). Therefore, the therapeutic efficacy of the combinatorial blockade of these signaling cascades was assessed in these cells. Here the combined treatment with PI3K/mTOR inhibitor NVP-BEZ235 and MEK1/2 inhibitors UO126 or SL327 results in suppression of the level of both phospho-ERK and phospho-Akt and in significant reduction of sphere-forming ability, which indicates the impairment of self-renewal ability of CSCs (76). Moreover, the combination induces a higher expression level of the neural marker βIII-tubulin, indicating that the inhibition of those pathways promotes GBM CSCs neural differentiation (76). In *in vivo* xenograft animal models, the GBM CSCs treated with both NVP-BEZ235 and SL327 inhibitors form smaller tumors than GBM CSCs treated with the single agent, with a significant improvement of mice survival (76).

AZD2014 is an inhibitor of mTORC1 and mTORC2 complexes as demonstrated by its ability to decrease the phosphorylation of S6k and 4EBP1, markers of mTORC1 activity, and of AKT, a marker of mTORC2 activity (77). Although AZD2014 alone has slight effect on survival of GBM CSCs, the combination of AZD2014 and radiation decreases the survival of GBM CSCs, thus indicating that the dual inhibition of mTORC1 and mTORC2 complexes sensitizes GBM CSCs to radiotherapy. Mechanistically, AZD2014-induced GBM CSCs radiosensitization involves the impairment of the repair of radiation-induced DNA double strand breaks. Moreover, the combinatorial treatment significantly increases the survival of mice bearing GBM CSCs xenograft brain tumor compared to control and single treatments mice (77).

PI3K signaling cascade is commonly hyperactive in GBM multiforme, which is also characterized by the dysregulation of apoptotic pathway due to the high expression level of anti-apoptotic Bcl-2 family of proteins. Therefore, combined blockade of PI3K pathway and Bcl-2 family of proteins may represent a reasonable therapeutic strategy. With this aim, ABT-263 (Navitoclax), an inhibitor of Bcl-2 and Bcl-xL, was used in combination with GDC-0941, a PI3K inhibitor, for the therapeutic treatment of GBM cells and CSCs (78). On a mechanistic point of view, ABT-263 binding to Bcl-2 and Bcl-xL proteins prevents their interaction with pro-apoptotic Bcl-2 family members, which results in the activation of intrinsic apoptotic cascade. However, ABT-263 poorly inhibits Mcl-1, another member of Bcl-2 family of proteins with anti-apoptotic properties, which confers resistance to ABT-263. The combination of GDC-0941 and ABT-263 significantly reduces the viability of GBM cell lines and the neurosphere formation ability of GSCs compared to single treatments. These data provide evidence that combinatorial treatment not only affects the bulk of the tumor, but also targets the CSCs population within malignant gliomas (78). On a mechanistic point of view, the combination causes a loss in mitochondrial membrane potential, the activation of both initiator caspase-9 and effector caspase-3 and the apoptosis of both GBM cell lines and glioma CSCs. Moreover, Akt pathway induces BAD phosphorylation, thereby inhibiting its pro-apoptotic properties. GDC-0941 dramatically reduces the phosphorylation of AKT and pro-apoptotic BAD proteins and lowers Mcl-1 levels by enhancing its degradation through the proteasome in GBM cell lines and glioma CSCs. In summary, GDC-0941-mediated dephosphorylation of BAD and Mcl-1 reduction render GBM cell lines and glioma CSCs more sensitive to the apoptotic properties of ABT-263 (78).

Some combinatorial therapeutic avenues in GBM CSCs treatment are based on the targeting of molecules specifically expressed by GBM CSCs including PCNA-associated factor (PAF) and bone marrow and X-linked (BMX) nonreceptor tyrosine kinase. PAF is overexpressed in breast cancer and GBM CSCs and controls cancer cell stemness *via* Wnt signaling (79, 80). In addition, PAF promotes CSCs resistance to radiotherapy by interacting with PCNA and regulating PCNA-associated DNA translesion synthesis (79). PAF depletion in combination with radiation compromises self-renewal and radioresistance of GSCs, thus suggesting a new therapeutic approach for GBM treatment (79). In addition, the high expression level of PAF in breast CSCs opens the possibility to design new therapeutic approaches. About the 90% of human GBMs shows high level of expression and activation of BMX (81). Interestingly, BMX is overexpressed in GSCs compared to non-stem tumor cells and neural progenitor cells. The binding of interleukin-6 to gp130 receptor stimulates the BMX mediated-hyperactivation of STAT3 signaling pathway, which in turn promotes the maintenance of GSCs self-renewal and tumorigenic potential (81, 82). In addition, BMX contributes to chemotherapy and radiotherapy cancer cells resistance (83, 84). Ibrutinib inhibits the tyrosine kinase activity of BMX, reduces GSCs proliferation, suppresses GSCs self-renewal potential and tumor sphere formation, and induces GSCs apoptosis (81). *In vivo*, ibrutinib suppresses the tumor growth in mice bearing GSCs-derived orthotopic xenografts, thus improving the mice survival (81). Interestingly, ibrutinib does not affect the ability of neural progenitor cells to form neurosphere; moreover, it selectively targets stem-like glioma cells expressing BMX in GBMs xenograft mice with small impact on neural progenitor cells lacking BMX expression (81). On a molecular point of view, in GSCs ibrutinib treatment strongly reduces the level of active phosphorylated BMX, and in turn the level of active phosphorylated STAT3, thus resulting in the reduction of the expression of STAT3 targets genes (*Nanog* and *Oct4*). This effect is not observed on STAT3 activation in ibrutinib treated neural progenitor cells, further confirming the specificity of ibrutinib on GSCs (81). Based on these data, the therapeutic efficacy of combined ibrutinib and irradiation treatment was analyzed in mice bearing GSCs-derived tumors (81). Here the ibrutinib and irradiation combination results in a significantly more efficient inhibition of tumor growth and in a longer survival extension compared to ibrutinib and irradiation single agent treatments. This improved therapeutic efficiency of combined treatment is due to a decreased cell proliferation and an increased apoptosis of tumor cells in GSCs-derived xenografts (81).

Diffuse intrinsic pontine glioma (DIPG) is a brainstem pediatric tumor characterized by high molecular heterogeneity (85–87) that leads to classification in cohorts according to high expression of MYCN and Shh or to presence of *H3F3A* mutations (86, 88). DIPG is characterized by hyperactivation of Notch, therefore the administration of Notch inhibitor has been evaluated as a potential therapeutic approach. A sequential combinatorial therapy has been assessed including the MYCN inhibitor JQ1 and the GSI MRK003 with the aim to reduce tumor growth and the resistance to radiotherapy (89). Combined treatment of DIPG cell lines (JHH-DIPG and SU DIPGXIII) shows a reduced cell proliferation with increased apoptotic cell death in a shorter time frame respect to single drug administration. On the other hand, DIPG SF7761 cells are more resistant to combinatorial treatment, even though comparative analyses at molecular level show a general downregulation of MYCN and MYC proteins levels in all the 3 cell lines tested, thereby suggesting that the molecular heterogeneity of DIPG plays an important role in the efficacy of therapy.

A summary of the preclinical studies performed on glioma CSCs is provided in **Table 2**.

**Table 2:** Combinatorial therapies tested in pre-clinical studies to treat glioma CSCs.

Tumor	Combinatorial therapy	Molecular target	Phenotypic outcome	Cellular/animal models	References
Glioma	SANT-1 + Guggulsterone	Smo + Ras/NFkB	Reduction of CSCs viability	Glioma CSCs and non-stem cells	(61)
Glioblastoma	Temozolomide + demethoxycurcumin	STAT3	Inhibition of glioma stem cells proliferation, G0/G1 cell cycle arrest and apoptosis induction	Glioma CSCs	(63)
Glioblastoma	Tipifarnib + RO4929097	Cell cycle	CSCs death	CD133^+^ CSCs	(68)
		Progression factors + Notch	Reduction of tumor growth	GBM mouse model	
Glioblastoma	LY2109761 + radiation	TGF-β type I receptor	Reduction of CSCs self-renewal and proliferation; induction of apoptosis	GBM cell lines and GSCs	(75)
			Delay of tumor growth and increase of mice survival	GBM subcutaneous and GSCs orthotopic xenograft tumor models	
Glioblastoma	UO126/SL327 + NVP-BEZ235	MEK1/2 + PI3K/mTOR	Reduction of CSCs sphere-forming ability, induction of CSCs neural differentiation	GBM CSCs	(76)
			Inhibition of tumor growth, increase of mice survival	Xenograft animal models	
Glioblastoma	AZD2014 + radiation	mTORC1 and mTORC2	Decrease of CSCs survival	GBM CSCs	(77)
			Increase of mice survival	GBM CSCs xenograft mouse model	
Glioblastoma	GDC-0941 + ABT-263	PI3K + bcl-2 and Bcl-xL	Decrease of CSCs neurosphere formation ability, induction of CSCs apoptosis	GBM cells and CSCs	(78)
Glioblastoma	PAF shRNA + radiation	PAF	Decrease of GSCs self-renewal and radioresistance	Glioma stem cells	(79)
Glioblastoma	Ibrutinib + radiation	BMX	Decrease of GSCs proliferation, suppression of self-renewal and tumor sphere formation, Induction of apoptosis	GBM CSCs	(81)
			Suppression of tumor growth, increase of mice survival	Mice bearing GSCs-derived orthotopic xenografts	
Pediatric diffuse intrinsic pontine glioma	MRK003 + JQ1	Notch + MYCN	Suppression of CSCs proliferation	JHH-DIPG and SU DIPGXIII cell lines	(89)

#### Medulloblastoma

Medulloblastoma is a brain tumor which occurs exclusively in the posterior fossa. Evidence suggests that PI3K and MAPK are reciprocal bypass pathways that can promote resistance to drugs targeting either pathway alone (35). Therefore, the dual inhibition of these pathways could represent a new therapeutic approach. Frank Eckerdt and collaborators demonstrated that the pharmacological blockade of MNK sensitizes medulloblastoma CSCs to targeted PI3Kα inhibition (90). *In vitro* the combined MNK and PI3Kα targeting significantly impairs the neurosphere growth compared to single treatments, and in *in vivo* xenograft animal models it reduces tumor growth and prolongs the mice survival compared to single treatments (90).

A summary of the preclinical study performed on medulloblastoma CSCs is provided in **Supplementary Table 1**.

#### Neuroblastoma

Neuroblastoma is an extracranial solid tumor of the sympathetic nervous system. Kwang Woon Kim and collaborators (91) showed that the activation of both AKT2/mTOR and MAPK signaling pathways in neuroblastoma cells is associated with the acquisition of cancer stem like phenotype (characterized by the expression of CD133, SOX2, ALDH, Nestin, Oct4, and Nanog stem cells markers and increased sphere-forming ability) and cisplatin- and radiation- resistance. The blocking of these signaling cascades with specific inhibitors of AKT2 (CCT128930) and MEK (PD98059) results in significantly higher reduction of sphere formation, cell proliferation, and cell migration compared to the single treatments in these cisplatin- and radiation- resistant cells, thus demonstrating the therapeutic efficacy of combined treatment to fight neuroblastoma CSCs (91).

A summary of the preclinical study performed on neuroblastoma CSCs is provided in **Supplementary Table 1**.

### Breast Cancer

The combined therapeutic strategies investigated in breast CSCs treatment are based on the combination of chemotherapeutic agents with molecular targeted therapies.

EW-7197 is TGF-β type I receptor kinase inhibitor which has been proved to be efficient in inhibiting the paclitaxel-induced CSCs properties in breast cancer cell lines and xenograft animal models (92). Paclitaxel treatment induces the increase of intracellular ROS, that in turn promote the EMT (93) through the *Snail* increased expression (92). The EMT increases the population of CSCs, as exemplified by the enhanced expression of pluripotency regulators (*Oct4*, *Nanog*, *Klf4*, *Myc*, and *Sox2*) and stemness markers [CD44^+^/CD24^-^ ratio, aldehyde dehydrogenase 1 (ALDH1)] and increased mammosphere-forming efficiency (92). The combinatorial treatment of EW-7197 and paclitaxel suppresses both *in vitro* and *in vivo* paclitaxel-induced ROS increase, Snail expression, EMT, and cancer stem-like properties (exemplified as reduction of the expression of CSCs markers and pluripotency regulators). Furthermore, it improves the therapeutic effect of paclitaxel by decreasing the lung metastasis and increasing the survival time *in vivo* (92).

The chemotherapy-induced CSCs enrichment in triple negative breast cancer (TNBC) is mediated by the reciprocal regulation of ERK and p38 activities (94), thus suggesting that the dual targeting of these pathways could represent a new therapeutic strategy to fight breast CSCs. Driven by this hypothesis Lu and collaborators (94) showed that the pharmacological inhibition of p38 activity by SB203580 blocks paclitaxel-induced expression of Nanog, Sox2, and Klf4 pluripotency factors and abrogates the paclitaxel-induced increase in the percentage of CSCs. Moreover, the combination of paclitaxel and p38 inhibitor LY2228820 significantly affects the tumor grow of mammary fat pad xenograft animal models compared to either drug alone. LY2228820 abrogates paclitaxel-induced increase in CSCs and mammosphere-forming cells and expression of Nanog and Klf4 stemness factors, indicating efficacy of p38 inhibitors, in combination with chemotherapy, in the eradication of breast CSCs (94).

The combination of src inhibitor saracatinib and gemcitabine sensitizes gemcitabine-resistant triple negative breast CSCs, overexpressing Src and its active form p-Src, to gemcitabine through the down-regulation of AKT/c-Jun pathway (95). This combination dramatically reduces the viabilities, survival and colony formation of gemcitabine-resistant breast CSCs compared to single agents (95). Moreover, it increases the expression of pro-apoptotic protein BAX and decreases the level of anti-apoptotic proteins Bcl-xL and Survivin, thus resulting in an enhancement of gemcitabine-induced apoptosis in gemcitabine-resistant breast CSCs than the single treatments (95). In addition, the combinatorial treatment reduces the expression of migration associated proteins p-FAK and MMP-3, thus resulting in impairment of gemcitabine-resistant breast CSCs migration compared to single treatments. Src inhibition combined to gemcitabine dramatically impairs the ability of sphere forming and the expression of CSCs markers, such as CD44 and Oct-4, compared with either agent alone, reflecting the synergistic inhibition of breast cancer stemness (95).

A further study shows the efficacy of combined therapy relying on the pre-treatment with selective WNT antagonists vantictumab (OMP-18R5) or ipafricept (OMP-54F28) followed by the taxane plaxicitin administration (96). Vantictumab (OMP-18R5) and ipafricept (OMP-54F28) impair respectively the binding of WNT to FZD receptors 1,2,5,7 and 8 (97) and to FZD8-Fc, a fusion protein between the extracellular ligand binding domain of FZD8 receptor and Fc domain of human immunoglobulin G1 (IgG1) (97, 98). It is worthy to mention that these antagonist compounds in combination with taxane plaxicitin act synergistically displaying anti-tumor activity on different type of tumor cells by using PDX models such as breast, ovarian and pancreatic cancers (96). In detail, the pre-administration of WNT antagonists sensitizes CSCs to plaxicitin therapy leading to potentiate the drug induced-mitotic cell death (mitotic catastrophe) in tumor cells responsive to the treatment and to reduce the number of CSCs.

In a rare subset of breast CSCs, characterized by high expression of αvβ3 and Slug and low expression of PUMA (αvβ3^+^/Slug^+^/PUMA^Lo^), Qi Sun and collaborators identified a signaling pathway, consisting of the integrin αvβ3, Src and the transcription factor Slug, responsible for the down-regulation of the expression of the pro-apoptotic molecule PUMA, which results in promoting the survival of breast CSCs and tumor aggressiveness, independently of hormone receptor status or molecular subtype (99). The genetic (by shRNA) or pharmacological (by dasatinib or saracatinib) blockade of Src activity disrupts this signaling axis in CSCs and drives PUMA expression, which in turn leads to decreased mammosphere formation, self-renewal and tumor initiation. Therefore, Src inhibition specifically targets CSCs and decreases breast cancer metastasis *in vivo* (99). Src inhibition is effective in targeting CSCs in anchorage-independent conditions; however, it is relatively ineffective against adherent cells, suggesting the presence of innate resistance factors. Since pro-apoptotic PUMA binds to pro-survival Bcl-2 proteins, limiting its ability to initiate apoptosis, the authors hypothesized that Src inhibition resistance of breast CSCs is mediated by the interaction between PUMA and the pro-survival Bcl-2 family members and the combinatorial inhibition of Src and the appropriate pro-survival Bcl-2 factor should result in the overcoming breast CSCs resistance to Src inhibition, thus resulting in the further improvement of CSCs depletion. As assumed, the Src inhibitor resistance of CSCs is mediated by the interaction between PUMA and the pro-survival Bcl-2 and Bcl-xL factors. The clinically-approved Bcl-2 selective inhibitor venetoclax is able to prevent PUMA binding to Bcl-2, freeing PUMA to induce intrinsic apoptosis. Therefore, the combinatorial treatment with both venetoclax and dasatinib was investigated. This treatment increases the percentage of apoptotic CSCs compared to dasatinib alone treatment, thus resulting in a synergistic reduction of breast CSCs viability (100). In addition, it further reduces stemness properties, including self-renewal and tumorsphere formation, compared to Src inhibition alone (100).

Glutathione S-transferase omega 1 (GSTO1) plays a major role in detoxification of chemotherapeutic agents in cancer cells. Recently, Lu and collaborators showed that GSTO1 plays a role in specification of the breast CSCs phenotype in response to chemotherapy independently of its enzymatic activity (101). Here the exposure of breast cancer cells to chemotherapy induces HIF-dependent expression of GSTO1, which interacts with ryanodine receptor 1 (RYR1) to increase intracellular Ca^2+^ levels, which in turn activates the PYK2/Src/STAT3 signaling cascade leading to breast CSCs enrichment (101). Thus, it was hypothesized that the genetic (by siRNA) or pharmacological (by inhibitor sulfonamide chalcone S2E) inhibition of GSTO1 in combination with chemotherapy should sensitize the breast CSCs to chemotherapeutic treatment. Actually, combined treatment of GSTO1 inhibitor S2E or siRNA with tamoxifen significantly reduces cell viability, cell migration and mammospheres forming efficiency of breast CSCs compared to single treatments, while increases the breast CSCs apoptosis (102). On the mechanistic point of view, GSTO1 inhibition leads to decreased activation of pro-migration and pro-survival ERK1/2, AKT, p38, Src and STAT3 signaling pathways and drives the activation of the stress kinase JNK, that in turn induces the pro-apoptotic proteins BAX, cytochrome c and cleaved caspase-3 of the mitochondrial apoptosis signaling pathway (102).

A further therapeutic approach using chemotherapy in combination with antibody therapy against the CSCs-associated molecule Nodal was assessed in TNBC cellular models (103). Nodal, an embryonic morphogen of TGF-β superfamily, is a regulator of early embryonic development (103), is highly expressed in several aggressive neoplasms (104) and plays a role in tumor growth, metastasis, CSCs phenotype and resistance to conventional therapy (103, 104). Nodal high expression drives maintenance of self-renewal and is associated to stem cell markers expression in CSCs (105, 106), vice versa the Nodal signaling inhibition causes the reduction of tumorigenesis, metastasis, invasion, angiogenesis, and plastic stem cell phenotype in several cancer types (105, 107–109). Since Nodal is not typically expressed in most normal adult tissues, it represents a potential targetable CSCs-associated molecule. The *in vitro* sequential treatment with doxorubicin, an inducer of DNA damage, followed by anti-Nodal antibody regimen in TNBC cellular models, impairs the cellular stress (p38) and DNA repair (ChK1) pathways, thus resulting in significant cellular growth and viability decrease and significant cell apoptosis increase (103).

A summary of the preclinical studies performed on breast CSCs is provided in **Table 3**.

**Table 3:** Combinatorial therapies tested in pre-clinical studies to treat breast CSCs.

Tumor	Combinatorial therapy	Molecular target	Phenotypic outcome	Cellular/animal models	References
Breast cancer	EW-7197 + paclitaxel	TGF-β type I receptor	Inhibition of EMT and CSCs properties	Breast CSCs	(92)
			Inhibition of EMT and CSCs properties; reduction of metastasis; increase of survival	Xenograft mouse model	
Triple negative breast cancer	SB203580/LY2228820 + paclitaxel	p38	Inhibition of CSCs stemness factors expression, inhibition of CSCs expansion	Breast CSCs	(94)
			Inhibition of tumor growth	Xenograft animal models	
Breast cancer	Saracatinib + gemcitabine	Src	Reduction of viabilities, survival, colony formation, sphere forming ability and migration of CSCs	Gemcitabine-resistant breast CSCs	(95)
Breast cancer	OMP-18R5/OMP-54F28 + plaxicitin	FZD/FZD8-Fc + tubulin	Suppression of tumor growth	Patient-derived xenograft (PDX) models	(96)
Breast cancer	Dasatinib + etoposide	Src	Decrease of CSCs population	Breast CSCs	(99)
Breast cancer	Dasatinib + venetoclax	Src + Bcl-2	Induction of CSCs apoptosis, reduction of CSCs self-renewal and tumorsphere formation	Breast CSCs	(100)
Breast cancer	GSTO1 siRNA/S2E + tamoxifen	GSTO1	Decrease of cell viability, cell migration and mammospheres forming efficiency	Breast CSCs	(102)
Breast cancer	Doxorubicin + anti-Nodal antibody	Nodal	Decrease of cell growth and viability, induction of apoptosis	TNBC CSCs	(103)

### Prostate Cancer

Two combinatorial therapeutic approaches were investigated in preclinical studies for treatment of prostate CSCs.

Zhang and collaborators (110) provided a first evidence that the combinatorial treatment of Napabucasin (BBI608), an inhibitor of STAT3 approved for the treatment of metastatic colorectal carcinoma and pancreatic cancer, with chemotherapeutic agents could improve the chemotherapy efficacy by modulating the sensitivity of CSCs to the drugs. Here the treatment of PC3 prostate cancer cells with different concentrations of docetaxel in combination with Napabucasin results in more effective inhibition of cell proliferation compared to docetaxel alone, thus suggesting that napabucasin could significantly increase the sensitivity of prostate cancer cells to docetaxel by killing drug resistant CSCs.

L Chang and collaborators demonstrated that the radiotherapy resistance in prostate cancer is due to the activation of PI3K/Akt/mTOR signaling pathway, which promotes both the EMT (as exemplified by increased cell migration/invasion, downregulation of epithelial marker and upregulation of mesenchymal markers) and the acquisition of CSCs phenotype (as exemplified by increased expression of CSCs markers and sphere formation ability) (111). The combination of inhibitor BEZ235 (targeting both PI3K and mTOR) with radiation exposure decreases the activation of PI3K/Akt/mTOR cascade (in terms of phosphorylation status of PI3K/Akt/mTOR signaling proteins such as p-Akt, p-mTOR, p-S6K, p-4EBP1), reduces EMT (in terms of increased expression of E-cadherin and decreased expression of N-cadherin, Vimentin, OCT3/4, SOX2 and αSMA), reduces the expression of CSCs markers (including CD44, CD44v6, CD326, ALDH1) and self-renewal proteins (such as Nanog and Snail), stimulates the apoptosis and decreases colony formation ability compared to single treatments, thus suggesting that the dual PI3K/mTOR inhibitor BEZ235 increases radiosensitivity of prostate CSCs (111).

A summary of the preclinical studies performed on prostate CSCs is provided in **Supplementary Table 1**.

### Non-Small-Cell Lung Cancer

The therapeutic efficacy of combination of Napabucasin with chemotherapeutic agents in treatment of prostate CSCs was further corroborated by the study of Lauren MacDonagh and collaborators who analyzed the therapeutic efficacy of Napabucasin-cisplatin combination in non-small-cell lung cancer (NSCLC) CSCs treatment (112). Here Napabucasin reduces the expression of stemness related genes (*Nanog*, *Oct-4*, *Sox-2* and *cMyc*) and CSCs associated genes (*CD133* and *ALDH1*), which results in decrease of CSCs population present in cisplatin resistant NSCLC subtypes (113). Moreover, the combined treatment significantly reduces the proliferation of cisplatin resistant NSCLC subtypes compared to cisplatin-only treated cells, thus suggesting that Napabucasin re-sensitizes the cells to the drug cytotoxic effects (113). In addition, the combined treatment significantly impairs the clonogenic survival of cisplatin resistant NSCLC subtypes compared to Napabucasin-only treated cells and simultaneously increases the percentage of cisplatin resistant apoptotic cells compared to cisplatin alone, thus demonstrating the potential benefit of combining Napabucasin with current chemotherapy drugs, such as cisplatin, to decrease NSCLC cell survival and as a means of overcoming cisplatin resistance (113).

A summary of the preclinical study performed on NSCLC CSCs is provided in **Supplementary Table 1**.

### Head and Neck Squamous Cell Carcinoma

Three combined approaches of molecular targeted therapies with chemotherapy were investigated in *in vitro* preclinical studies in treatment of head and neck squamous cell carcinoma (HNSCC) CSCs. The first strategy combines the p38 inhibitor SB203580 and chemotherapeutic agent cisplatin (114). Here the combined treatment results in a reduction of survival and colony forming ability, increased apoptosis and an impairment of DNA damage response and repair capacity compared to cisplatin alone treatment. This finding suggests that the inhibition of p38 sensitizes HNSCC cells toward cisplatin. Interestingly, SB203580-cisplatin combination significantly reduces CSCs markers expression and tumor spheroid formation compared to cisplatin treated cells, thus showing that the combinatorial treatment impairs CSCs maintenance (114).

The second approach combines the targeting of the stem cell factor BMI1 (B cell specific moloney murine leukemia virus insertion site 1 gene) with cisplatin. BMI1, a core component of the polycomb repressive complex 1, is upregulated in a variety of human cancers and contributes to CSCs self-renewal and chemoresistance (115). Cancer cells in HNSCC are efficiently targeted by cisplatin, while BMI1^+^ CSCs are not. Therefore, the combined administration of the BMI1 specific inhibitor PTC-209 and cisplatin fights both tumor cells and CSCs thus reducing the BMI1^+^ CSCs-mediated lymph node metastases (116).

The combination between dasatinib and the mithramycin analog EC-8042 was also investigated in the treatment of HNSCC CSCs (117). Hermida-Prado and collaborators showed that dasatinib and saracatinib, in monotherapy regimes, strongly reduce the phosphorylation levels of active Src and FAK, and inhibit EMT, through the up-regulation of epithelial marker E-Cadherin and concomitant down-regulation of mesenchymal markers vimentin and Snail, thus resulting in a significant inhibition of HNSCC cell migration and invasion. However, both dasatinib and saracatinib fail to eliminate CSCs-enriched tumorsphere cultures of HNSCC cells, as revealed by the significant increase in the expression levels of CSCs markers, including ALDH1A1, SOX2, Nanog1 and Oct4, thus suggesting that these drugs, used as single agents, strongly enhance CSCs properties (117). To counteract the pro-stemness activity of dasatinib, the authors assessed the effects of the dual treatment with dasatinib and EC-8042 on HNSCC cellular and xenograft animal models, based on the observation that a strong decrease of CSCs viability and CSCs-related markers expression was observed in HNSCC (117) and in other cancers (118) treated with the mithramycin analog EC-8042. As a result of the combinatorial treatment, the HNSCC cell invasion ability, the viability of CSCs-enriched tumorspheres and the expression of CSCs-related factors (including ALDH1, SOX2, Nanog, Oct4, c-Myc and Notch1) are significantly affected. On a molecular point of view, the dasatinib-EC-8042 treatment inhibits active Src phosphorylation, Src-dependent phosphorylation and activation of FAK, AKT and p44/42-MAPK and reduces the levels of the EC-8042 target SP1 (118). In *in vivo* HNSCC xenograft animal model, the dual treatment leads to a significant reduction of tumor growth with the concomitant improvement in mice survival when compared to vehicle and dasatinib alone treatments (117). Of note, the significant reduction in the percentage of Ki67-positive cells, the strong increase in the percentage of apoptotic cells and the remarkable decrease in the CSCs markers (such as ALDH1A1 and SOX2) expression level was observed in tumors from dasatinib-EC-8042 treated mice (117). Furthermore, while tumorsphere formation slightly increases in dasatinib-treated tumors, the tumorspheres-forming ability is significantly inhibited in dasatinib-EC-8042 treated tumors, thus providing evidence that the combined treatment effectively targets CSCs properties in *in vivo* HNSCC animal model. Taken together these findings demonstrate that EC-8042 counteracts the pro-stemness effects of dasatinib on HNSCC cellular and animal models and that the combined dasatinib-EC-8042 treatment benefits from the anti-proliferative, anti-invasive and anti-stemness functions provided by each compound without antagonizing each other.

A summary of the preclinical studies performed on HNSCC CSCs is provided in **Supplementary Table 1**.

### Colorectal Cancer

5-fluorouracil (5-FU) administration remains the most effective regimen for the cure of colorectal cancer-patients (119, 120), despite tumor relapse is encountered after discontinuation of the chemotherapeutic treatment (121). WNT/β-catenin pathway is activated upon 5-FU administration and is involved in the maintenance of colorectal cancer 5-FU-treated CSCs (122). On a mechanistic point of view, the drug modulates p53 activity, which in turn leads to the expression of WNT3 that is a positive regulator of Wnt/β-catenin signaling in colorectal cancer cell lines that harbor wild-type p53, thus contributing to relapse of the tumor (122). As such the administration of the WNT inhibitor LGK-974 combined with 5-FU to patient-derived tumor organoids and patient-derived tumor cells has revealed to be effective in suppressing tumor regrowth after discontinuation of treatment. The combined treatment induces a strong decrease in β-catenin levels, thus resulting in downregulation of WNT signaling pathway. To further determine the pathophysiological importance of WNT inhibition *in vivo*, the effects of 5-FU and LGK-974 combinatorial treatment were evaluated in 3 wild-type PDX mouse models. Both 5-FU monotherapy and combinatorial therapy with LGK-974 and 5-FU effectively reduce tumor growth, whereas co-treatment increases the sensitivity of tumors to 5-FU. Treatment with 5-FU alone, however, increases β-catenin and CSCs markers in the remaining tumor, while concurrent LGK-974 treatment effectively suppresses the effects of 5-FU on the levels of β-catenin and CSCs markers, thus preventing from the recurrence of the tumor. Taken together these findings prompt to consider WNT inhibition and 5-FU treatment as a strategy to overcome poor survival rates in colorectal cancer-patients.

Nautiyal and collaborators showed that the combination of the src inhibitor dasatinib and curcumin is effective in eliminating chemo-resistant colon cancer cells (123). The combined treatment strongly reduces the expression of CSCs markers (*ALDH*, *CD44*, *CD133*, *CD166*) in intestinal adenomas from APCMin^+/-^ mice, thus suggesting that the dual treatment decreases the CSCs population in adenomas (123). *In vitro* dasatinib-curcumin treatment acts synergistically to significantly inhibit the growth of oxaliplatin chemo-resistant colon CSCs, the colonosphere formation, invasion potential and the expression of CSCs markers (such as *CD133*, *CD44*, *CD166* and *ALDH*), thus indicating that it is highly effective in reducing the colon CSCs population and inhibiting CSCs stemness properties (123).

A summary of the preclinical studies performed on colorectal CSCs is provided in **Supplementary Table 1**.

### Hepatocellular Carcinoma

HCC, one of the most common cancers worldwide, is characterized by high biological, molecular and clinical heterogeneity. Among the genes whose regulation appears altered, *CDK1* has been reported to be up-regulated in 46% of HCC tumor tissues and to correlate with poor prognosis of survival. On a molecular point of view, the CDK1/PDK1/β-catenin axis activation is linked to EMT, which in turn leads to an increased capacity of migration. Therefore, the dual blockade of CDK1 and PDK1 may represent a valuable combinatorial therapeutic strategy for HCC CSCs targeting. The administration of the cyclin-dependent kinase inhibitor (CDKI) RO3306 in association with the TKI sorafenib has been found functional to target CSCs in PDX tumor models of HCC (124). The treatment of PDX tumors with RO3306, sorafenib or the combination of the two drugs determines the tumor growth suppression of 75%, 42% and 92% respectively. These results clearly underline the efficacy of the combinatorial therapy and the positive effect of pretreatment with the CDKI in sensitizing CSCs to sorafenib. In particular, western blot analyses reveal the synergistic effect of the drugs combined use in down-regulating the CDK1, PDK1, β-catenin protein levels with the concurrent decrease in the levels of several CSCs stemness proteins, such as Oct4, Sox2 and Nanog (124).

A summary of the preclinical study performed on HCC CSCs is provided in **Supplementary Table 1**.

### Ovarian Cancer

The ovarian cancer is a heterogenous disease in which a multiplicity of distinct malignancies shares a common anatomical site. The high-grade serous subtype predominates in the clinical setting and is responsible for the highest rate of mortality among all forms of ovarian cancer. The prolonged Src inhibitor saracatinib treatment of high-grade serous ovarian cancer cells generates saracatinib-resistant cells, in which the activation of epidermal growth factor receptor (EGFR), HER2/ERBB2 and Raf/MEK signaling kinases was observed (33). Moreover, high expression levels of Src and MAPK active phosphorylated forms were detected in high-grade serous ovarian cancer ALDH1^+^ CSCs. Therefore, the efficacy of combined Src and MEK inhibition with Src inhibitor saracatinib and MEK1/2 inhibitor selumetinib was investigated (33). The combinatorial treatment inhibits the EGFR-1 and EGFR-2–mediated bypass MEK/MAPK activation observed with saracatinib alone and effectively targets CSCs subpopulation of ovarian cancer. In *in vitro* experiments, the dual treatment reduces ALDH1^+^ CSCs population and sphere-forming cell amount more effectively than monotherapies. *In vivo*, it causes a significant inhibition of xenograft tumor growth compared to single drug treatments as a consequence of the drastic reduction of ALDH1^+^ CSCs population. In fact, tumors dissociated after combined therapy show a significant reduction in ALDH1^+^ CSCs population and sphere forming cells upon serial xenografting compared to tumors dissociated after monotherapies (33).

A summary of the preclinical study performed on ovarian CSCs is provided in **Supplementary Table 1**.

### Pancreatic Cancer

Pancreatic cancer remains a deadly disease with a very low 5-year survival due to disease recurrence even after surgical resection and/or chemotherapeutic regimes. Therefore, the combinatorial therapy may represent a valuable opportunity. The combination of molecular targeted therapies with chemotherapeutic drugs was investigated in preclinical studies to fight pancreatic CSCs. Duong and collaborators showed that dasatinib sensitizes pancreatic CSCs to gemcitabine (125). In fact, dasatinib-gemcitabine treatment significantly decreases both Src and STAT3 activation (in terms of level of phospho-Src and phospho-STAT3), and the ALDH1A1 level, which in turn promotes the inhibition of CSCs proliferation and survival by the induction of apoptosis through activation of caspase-3/7 and PARP cleavage (125).

Similarly, the inhibition of Nodal signaling in combination with the chemotherapeutic agent gemcitabine induces the apoptosis of pancreatic CSCs, suppresses cells in S phase and *in vivo* tumorigenicity, thus suggesting that Nodal signaling inhibition reverses the chemoresistance of the tumorigenic CSCs population (105). In pancreatic cancer xenograft athymic mouse model, established by the implantation of pancreatic cancer cell line, the combination therapy significantly delays tumor growth and increases the mice survival compared to monotherapies (105). However, engrafted primary human pancreatic cancer tissue with a substantial stroma shows no response to combinatorial therapy, probably due to limited drug delivery. To improve the Nodal inhibitor delivery, a triple combination therapy, in which gemcitabine plus Nodal signaling inhibitor treatment was combined to Shh pathway inhibition, was used. This triple therapy results in the impairment of *in vivo* tumor growth and increase of long-term progression-free survival (105).

A summary of the preclinical studies performed on pancreatic CSCs is provided in **Supplementary Table 1**.

### Melanoma

A therapeutic approach of combination of the anti-Nodal antibody with the chemotherapeutic agent dacarbazine (DTIC) was tested in melanoma cell lines (107). Here, while the DTIC alone treatment induces an increase in cell population expressing Nodal, the sequential treatment with DTIC and anti-Nodal antibody exhibits a striking decrease in the viable cell population and a striking increase in the proportion of programmed cell death. Moreover, the combined treatment results in a significant reduction in cell invasion, thus suggesting that targeting Nodal impairs the invasive ability of DTIC-resistant melanoma cells (107). More interestingly, the combined DTIC and anti-Nodal antibody exposure significantly suppresses the cell proliferation and induces the apoptosis in multicellular tumor spheroid culture (107). Similarly, the treatment of melanoma cell line harboring the active V600E mutation of B-RAF, which is constitutively active in approximately half of the melanoma patients, with B-RAF inhibitor RG7204 (vemurafenib) drives the selection of B-RAF inhibitor-resistant cells. These cells further treated with anti-Nodal antibody exhibit a marked decrease in viability and dramatic increase in cell death, thus showing that Nodal expression maintained in B-RAF inhibitor-resistant cells is also targetable with anti-Nodal antibodies with a significant benefit in therapeutic efficacy of melanoma treatment (107). The efficacy of combination of anti-Nodal antibody and dabrafenib, another inhibitor of B-RAF, was further investigated. Here *in vitro* and *in vivo* experiments were carried out on highly metastatic melanoma cell line and xenograft mice model with active B-RAF (V600E) mutation (126). The combined treatment significantly reduces the Nodal expression, the *in vitro* anchorage-independent colony formation and tumorigenic growth potential, and the *in vivo* lung metastases compared to the single treatments (126).

A summary of the preclinical studies performed on melanoma CSCs is provided in **Supplementary Table 1**.

### Esophageal Cancer

The therapeutic efficacy of the combined targeting of Heat shock protein 90 (Hsp90) and STAT3 was investigated in treatment of esophageal CSCs. Hsp90 is a molecular chaperone which binds to its client proteins to stabilize them and assist in their folding. It is a positive modulator of prostate CSCs, in fact it upregulates stemness markers, promotes self-renewal, and enhances tumor sphere growth (127). Hsp90 inhibition is effective in targeting CSCs in several cancers (127–130). One of the client proteins of Hsp90 is STAT3. The association between Hsp90 and STAT3 is necessary for STAT3 phosphorylation, dimerization, and nuclear translocation (131). This evidence suggests the combinatorial inhibition of Hsp90 and STAT3 as a therapeutic approach in CSCs treatment. To this aim the therapeutic efficacy of the combined treatment of Hsp90 inhibitor SNX-2112 and the sh-mediated knockdown of STAT3 was assessed in esophageal cancer stem-like cells (ECSLCs). Here the dual treatment strongly inhibits the proliferation of ECSLCs, induces G2/M phase arrest and apoptosis of ECSLCs, significantly decreases the colony formation ability and the colony size of ECSLCs compared with shSTAT3 and SNX-2112 alone (132). On a molecular point of view, the combined treatment reduces the levels of phosphorylation of Hsp90 client proteins involved in cell proliferation including p-p38, p-JNK and p-ERK, decreases the mRNA level of adenosine triphosphate-binding cassette transporter super-family *ABCB1* and *ABCG2* and the expression level of pro-survival Bcl-2 protein, and increases the expression level of the pro-apoptotic Bax protein compared with SNX-2112 and shSTAT3 alone groups (132). In addition, the combined treatment significantly reduces the tumor growth in *in vivo* ECSLCs xenograft tumor models compared to single treatments (132).

A summary of the preclinical study performed on ECSLCs is provided in **Supplementary Table 1**.

## CSCs-Centered Combinatorial Strategies: Which are the Concluding Remarks?

The combinatorial strategies herein reported foresee the targeting of developmental and oncogenic signaling pathways that are key players in defining several features of CSCs biology, including self-renewal, stemness, EMT, CSCs dormancy, as well as drug resistance, radio-resistance, tumor initiation and dedifferentiation, widely discussed in many published reviews (17–28).

The molecular mechanisms underlying the combinatorial strategies have been described in detail for each approach (please refer to the text above). In spite of the multiplicity of the strategies, some considerations can be taken into account to better address some common aspects of the topic.

Most of the dual therapies are based on the combination of molecular targeted therapies (for details the reader is referred to **Tables 1** **–** **3** and **Supplementary Table 1**). The rationale underlying this combination is based on the evidence of the activation of bypass pathway(s) and the feedback loops and cross-talk occurring. For the sake of simplicity, the following cases are presented as examples. The extensive cross-talk between MAPK and PI3K/AKT/mTOR pathways limits the therapeutic efficacy of the monotherapeutic regimes which target one or the other pathway (29, 30, 34, 35). The combinatorial strategies here reviewed provide a proof of concept that the dual blockade of these pathways by different combinations of molecules as diverse as alpelisib-CGP57380 (90), CCT128930-PD98059 (91) and UO126/SL327-NVP-BEZ235 (76) allows to overcome the activation of bypass pathway, thus resulting in the stronger therapeutic efficacy in CSCs eradication. Similarly, the Src inhibition induced-activation of MAPK signaling cascade (31–33) is overcome by the saracatinib-selumetinib dual treatment (33). The activation of STAT3 signaling pathway upon TKI-mediated BCR-ABL1 inhibition suggests the combinatorial STAT3 and BCR-ABL1 targeting as a new therapeutic avenue to eradicate CML stem and progenitor cells (57, 58). In addition, the concomitant activation of more than one signaling cascade represents the basis for the molecular targeted combinatorial approaches as demonstrated in the case of the combination of the β-catenin/CBP antagonist C-82 and the FLT3-TKI sorafenib or quizartinib in LSC treatment (36).

Other strategies combine the blockade of the anti-apoptotic Bcl-2/Bcl-xL proteins and MAPK or PI3K or Src signaling cascades by the administration of cobimetinib-venetoclax (46), GDC-0941-ABT-263 (78) and dasatinib-venetoclax 100] respectively. In these cases, the rationale for these approaches relies on the ability of MAPK, PI3K and Src signaling to negatively regulate the apoptotic pathway. Indeed, MAPK stabilizes anti-apoptotic Mcl-1 and inactivates pro-apoptotic BIM (44, 45), PI3K stabilizes Mcl-1 and inhibits the pro-apoptotic properties of BAD (78), Src down-regulates pro-apoptotic PUMA (100), thus promoting CSCs survival (for more details see the text). Therefore, the apoptosis is strongly stimulated by dual inhibition treatment rather than single treatment regimes.

Related to combinatorial strategies including chemotherapeutic drugs, their use as single agents in cancer treatment activates signaling pathways that promote the enrichment in CSCs population. This is the case of paclitaxel and gemcitabine which induce the activation of p38 and Src signaling respectively, thus resulting in CSCs increase (94, 95). This mechanism of action justifies the dual combination of SB203580 or LY2228820 with paclitaxel (94) and the dual combination of saracatinib with gemcitabine (95) as new therapeutic avenues to fight CSCs. Similarly, the ability of 5-FU to activate Wnt signaling cascade represents the rationale for the dual 5-FU/LGK-974 treatment as a new approach in CSCs therapy (122). Accordingly, the combinatorial strategies based on the use of chemotherapeutic agents in combination with inhibitors of specific signaling pathways have been developed to overcome this drawback and to foster the effect of chemotherapy. Examples are provided by the combination of chemotherapeutic drugs, interfering with DNA-related physiologic functions, such as mitosis (paclitaxel, docetaxel), DNA synthesis (cytarabine (Ara-c), etoposide, gemcitabine, daunorubicin, doxorubicin), DNA repair (cisplatin) or DNA transcription (EC-8042), with the inhibitors of different signaling pathways (including Wnt, TGF-β, MAPK, STAT, Sonic Hedgehog, Src, PI3K/AKT/mTOR) playing key roles in CSCs survival, proliferation, self-renewal and EMT (42, 49, 92, 94–96, 110, 112–114, 117, 122, 125).

On the same line of evidence, the radiotherapy induces the activation of signaling pathways, such as TGF-β and PI3K/AKT/mTOR, which promote EMT and CSCs stemness. Therefore, the radiotherapy regime combined with the inhibition of these signaling cascades represents the rationale behind few therapeutic strategies herein described, such as radiation-LY2109761 (75), radiation-AZD2014 (77), radiation-BEZ235 (111) (for details the reader is referred to the text).

## Discussion

**Figure 2:**
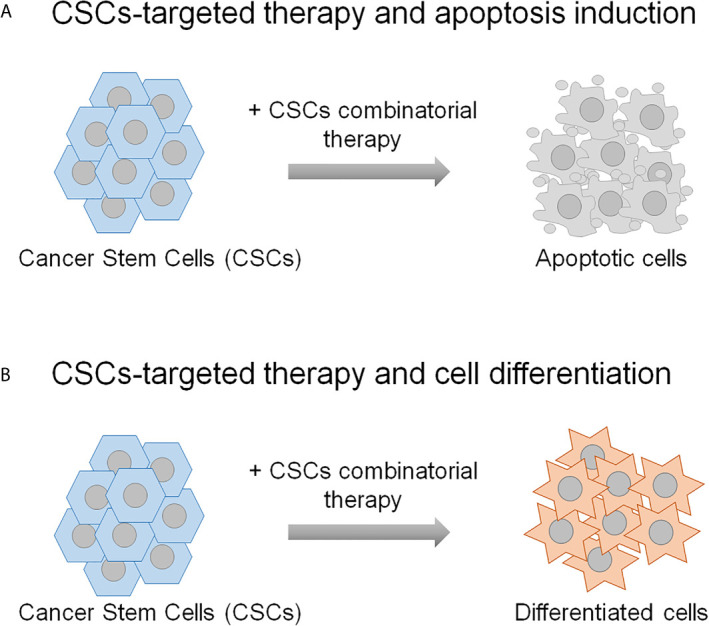
The schematic representation shows the mechanisms underlying the CSCs combinatorial strategies applied in the preclinical studies described in the text. CSCs-targeting can result in their eradication **(A)**, or differentiation **(B)**. The remaining non-stem tumor cells are thought to be sensitive to the conventional chemo- and radio-therapies.

Accumulating evidence from studies in cancer shows that the CSCs, although constitute a rare cell subpopulation within the tumor bulk, represent the major cause of cancer therapy failure and tumor relapse. Therefore, these cells have become attractive candidate for preclinical studies to define promising targeted therapies in the treatment of cancer. Indeed, different CSCs-centered anti-cancer strategies have been assessed that foresee the targeting CSCs specific surface markers and tumor microenvironment, the inhibition of ATP-binding cassette transporters, the switching off CSCs self-renewal and survival signaling pathways and immunotherapy (133, 134). In addition, more recent developments include nano-drug delivery systems, mitochondrion targeting, autophagy and hyperthermia (134). Herein, the description of the combinatorial strategies between the molecular targeted therapy and the chemotherapy or the radiotherapy to fight CSCs is reviewed. The preclinical studies presented in this review demonstrate that the therapeutic strategies based both on the combination of two molecular targeted therapies and on the combination of molecular targeted therapy with chemotherapy or radiotherapy result in a more effective targeting of CSCs compared to the single treatments. The most common mechanism through which these dual strategies affect CSCs relies on their eradication due to the induction of the apoptosis (**Figure 2A**), although a case of induction of CSCs differentiation is also reported (76) (**Figure 2B**). On a biological point of view, these combinatorial approaches impair some distinguishing features of CSCs, including proliferation and viability, self-renewal, clonogenic and sphere-forming potential and anti-apoptotic pathway activation, thus resulting in the reduction of CSCs radio- and chemo-resistance (the reader is referred to **Tables 1** **–** **3** and **Supplementary Table 1**).

The preclinical studies herein described have been performed on some hematological malignancies, including the AML and CML, and on some solid tumor types, mainly tumors of nervous system and breast cancers. Few studies of combinatorial avenues have been carried out on other cancers, including HCC, prostate cancer, pancreatic cancer, NSCLC, HNSCC, colorectal cancer, melanoma and ovarian cancer. The promising results achieved by the CSCs-treatment of these types of tumors strongly suggest to further extend the combinatorial therapeutic approaches to the treatment of additional cancers. In addition, preclinical studies demonstrate that some therapeutic combinations selectively affect CSCs and non-stem tumor cells without impact on normal stem cells, thus further supporting them as new therapeutic avenues (46, 58, 61, 81). Of note, the preclinical studies here presented show that some Food and Drug Administration (FDA)-approved drugs/inhibitors (such as Napabucasin, dasatinib, tipifarnib, venetoclax and sorafenib), already used in clinic for cancer treatment, in combination with clinically-approved chemotherapeutic agents (including cisplatin, paclitaxel, 5-FU, docetaxel) or radiation regimes effectively affect CSCs from different tumors. Among these, Napabucasin, a STAT3 inhibitor approved for the treatment of metastatic colorectal carcinoma and pancreatic cancer, is an example. In combination with chemotherapeutic drugs (docetaxel or cisplatin) it sensitizes prostate and NSCLC CSCs to chemotherapy. Taken together these achievements suggest that the therapeutic combinatorial strategies can be translated to clinical trials.

However, it is worthy to mention that some FDA-approved drugs/inhibitors, such as dasatinib and saracatinib, with proven powerful anti-tumor properties in preclinical studies, have shown limited clinical efficacy once translated in cancer patient’s treatment (135–139). Of note, these two drugs enhance the CSCs properties in the HNSCC cellular models (117) and, more importantly, dasatinib fails to eliminate CSCs-enriched tumorspheres or to impair tumor growth in HNSCC xenograft models (117). On the same line of evidence, dasatinib worsens the effect of cetuximab in combination with radiotherapy in HNSCC xenograft models (140) and saracatinib does not affect tumor growth but impairs invasion and lymph node metastasis in HNSCC xenograft model (141). In clinical settings, these two drugs do not demonstrate any significant effect as single agents in patients with operable, recurrent and/or metastatic HNSCC (142–144). The deleterious pro-stemness activities exerted by dasatinib and saracatinib in HNSCC cell lines could envisaged as the main cause for the lack of clinical efficacy in HNSCC patients when used in monotherapeutic regimes. Similarly, dasatinib in combination with quercetin worsens the progression of liver disease, in a mouse model of obesity- and age- dependent liver disease, which fully recapitulates the non-alcoholic fatty disease (NAFLD) and HCC (145). In contrast to these findings, dasatinib inhibits the expression of CSCs marker SOX2 and tumorsphere formation in NSCLC cells (146, 147). These findings clearly demonstrate the contradictory effects of dasatinib suggesting that the observed anti-CSCs therapeutic efficacy is tumor-dependent, and thus an adequate patient stratification is required to select the patients who may benefit from dasatinib treatment.

Although the combinatorial treatments are promising strategies for CSCs-targeted therapies, the molecular heterogeneity of tumors adds another layer of complexity. Indeed, the molecular heterogeneity requires the targeting of specific signaling molecules according to the dysregulated signaling pathways, as exemplified in the case of breast, glioma and AML CSCs for which more than one combinatorial approach has been proven to be successful in cancer treatment. Therefore, the understanding of molecular and signaling networks active in each tumor subtype and in related CSCs is a relevant issue to be addressed. The implementation of –omic techniques, contributing to dissect the molecular heterogeneity of the tumors, provides the tools for defining personalized-therapeutic treatments.

A further consideration regards the cellular and animal models used in the preclinical studies. In fact, the most of these studies have been performed *in vitro* on CSCs from established tumor cell lines or from patients’ tumor specimens. Given the roles played by tumor microenvironment in defining tumor and CSCs properties, more appropriate models should be developed in further studies, including PDXs and organoids, to investigate the therapeutic efficacy of combinatorial approaches. The relevance of tumor microenvironment in defining both the CSCs properties and the therapeutic efficacy of the anti-CSCs combinatorial therapies is highlighted by the triple therapy that combines the Shh pathway inhibitor with Nodal inhibitor and gemcitabine to impair the pancreatic PDX tumor growth *in vivo* (105). Similarly, Cazet and collaborators demonstrated that Hedgehog ligand-activated cancer-associated fibroblasts provide an environment that promotes the tumor cells to acquire a chemoresistant stem-like phenotype (148). The treatment with the SMO inhibitors, reverting the cancer-associated fibroblasts gene expression changes induced by Hedgehog signaling, sensitizes the tumors to docetaxel chemotherapy in TNBC cells- and patient-derived xenograft animal models, thus resulting in inhibition of tumor growth and increased mice survival (148). In addition, the SMO inhibitor-docetaxel combined treatment was effective in treating a proportion of women with metastatic disease who had previously failed on taxane chemotherapy (148). These findings are further corroborated by the recent study of Brown and collaborators (149) who performed an *ex vivo* translational study on tumors dissected from ovarian cancer patients treated with metformin, a modulator of cellular metabolism, and matched non-metformin-treated control patients. Their findings demonstrate not only that metformin-treated tumors exhibit a significant reduction in CSCs population and are more sensitive to cisplatin treatment *ex vivo* compared to non-metformin-treated tumors, but also that metformin may impair stemness indirectly *via* an impact on tumor microenvironment. Indeed, metformin induces epigenetic changes in the cancer-associated mesenchymal stem cells, which prevents the ability of these cells to drive the chemoresistance *ex vivo* (149).

Drug specificity, selectivity and the related drug treatment side-effects are additional issues to be considered in designing new therapeutic avenues. For example, GSIs are not selective for Notch given that they may target a variety of γ-secretase substrates as well with different cellular outcomes (150). In addition, clinical trials using GSIs frequently counted drug-related side-effects (151). Therefore, there is an urgent need to develop more selective, potent and healthy drugs. For such a purpose, since several FDA-approved drugs have repurposing effects, they could constitute a further repertoire of potential anti-cancer agents. Indeed, one of the most promising repurposing approaches relies on the administration of antibiotics in combination with chemotherapy. In line with this approach, linezolid, a bactericidal antibiotic with the ability to induce a block in protein translation at mito-ribosomes with consequent alteration of mitochondrial functions, has been recently proposed in combination with the autophagy inhibitor HCQ as valuable strategy in TNBC anticancer therapy (152). Of note, concomitant treatment with linezolid and HCQ reduces tumor size in TNBC xenograft mice and colony formation in parental TNBC cells, and corresponding chemoresistant cells and CSCs compared to the linezolid alone treatment (control) (152). Similarly, a recent clinical pilot study was performed to assess the efficacy of antibiotic treatment in anticancer strategy (153). Given the important role of mitochondrial metabolism in CSCs, the FDA-approved doxycycline inhibitor was administered in short term pre-operative treatment (14 days before surgery) with the aim to follow the CSCs reduction in a small group of early breast cancer patients. The expression of known biomarkers of stemness, mitochondria, cell proliferation, apoptosis and neo-angiogenesis was analyzed in pre-operative specimens and matched surgical specimens. After doxycycline treatment, surgical tumor samples showed a significant decrease in the expression level of the stemness markers CD44 and ALDH1 respect to matched pre-operative and pre-doxycycline samples. Differently, the levels of markers of mitochondria, proliferation, apoptosis, and neo-angiogenesis were found similar in all the analyzed specimens (153). Although a further clinical study involving a larger number of patients should be performed to validate these preliminary data, this small-scale clinical study suggests that the mitochondrial functions inhibition may represent a new potential strategy for the CSCs eradication. In addition, the application of the Artificial Intelligence (154), a new emerging technology in drug discovery field, will implement the number of candidate molecules to be screened in order to design new combinatorial therapeutic avenues.

Taken together the combinatorial strategies described herein represent a starting point for future studies to design CSCs-targeted and personalized therapies.

## Author Contributions

GC and DS discussed, wrote, and approved the manuscript. AC discussed and approved the manuscript. All authors contributed to the article and approved the submitted version.

## Funding

This article was funded by Italian Association for Cancer Research (AIRC, Milan, Italy), IG 2017 id. 20095 to Antonino Colanzi.

## Conflict of Interest

DS is a topic editor of the research topic: Combinatorial approaches for cancer treatment: from basic to translational research.

The remaining author declares that the research was conducted in the absence of any commercial or financial relationships that could be construed as a potential conflict of interest.

## Publisher’s Note

All claims expressed in this article are solely those of the authors and do not necessarily represent those of their affiliated organizations, or those of the publisher, the editors and the reviewers. Any product that may be evaluated in this article, or claim that may be made by its manufacturer, is not guaranteed or endorsed by the publisher.

## Abbreviations

CSCs, Cancer stem cells; ALDH1, aldehyde dehydrogenase 1; AML, acute myeloid leukemia; CML, chronic myeloid leukemia; ROS, reactive oxygen species; EMT, Epithelial to Mesenchymal Transition; Shh, Sonic Hedgehog; TGF-β, transforming growth factor-beta; JAK, Janus kinase; STAT3, signal transducer and activator of transcription 3; PI3K, phosphatidylinositol 3-kinase; AKT, V-Akt murine thymoma viral oncogene; mTOR, mammalian target of rapamycin; MAPK, Mitogen-activated protein kinase; Src, V-SRC avian sarcoma; ERK, extracellular signal-regulated kinase; JNK, c-Jun N-terminal kinase; EGFR, epidermal growth factor receptor; MEK, mitogen-activated protein kinase kinase; LSCs, leukemia stem cells; TNBC, triple-negative breast cancer; HCC, hepatocellular carcinoma; PDX, patient-derived xenograft; GSIs, γ-secretase inhibitors; FTIs, farnesyltransferase inhibitors; GBM, glioblastoma; GSCs, glioma stem cells; MNK, MAPK-interacting kinase; mTORC, mTOR complex; NSCLC, non-small cell lung cancer; HNSCC, head and neck squamous cell carcinoma; TMZ, temozolomide; DMC, demethoxycurcumin; TKI, tyrosine kinase inhibitor; PUMA, p53-upregulated modulator of apoptosis; BMI1, B cell specific moloney murine leukemia virus insertion site 1 gene; PAF, PCNA-associated factor; BMX, bone marrow and X-linked; DTIC, dacarbazine; Hsp90, Heat shock protein 90; ECSLCs, esophageal cancer stem-like cells; GSTO1, Glutathione S-transferase omega 1; FDA, Food and Drug Administration; TNF-α, Tumor Necrosis Factor-α; IFNs, interferons.
