# Corrigendum: Combinatorial Strategies to Target Molecularand Signaling Pathways to Disarm Cancer Stem Cells

**DOI:** 10.3389/fonc.2021.749040

**Published:** 2021-08-16

**Authors:** Giuliana Catara, Antonino Colanzi, Daniela Spano

**Affiliations:** Institute of Biochemistry and Cell Biology, National Research Council, Naples, Italy

**Keywords:** cancer stem cells, cancer, combinatorial strategies, signaling pathways, molecular pathways

In the original article, we neglected to include the funder Italian Association for Cancer Research (AIRC, Milan, Italy), IG 2017 id. 20095 to Antonino Colanzi.

## Addition of an Author

Antonino Colanzi was not included as an author in the published article. The corrected Author Contributions Statement appears below.

AC discussed and approved the manuscript.

With addition of Dr. Antonino Colanzi to the author list the Acknowledgement will be removed.

The authors apologize for these errors and state that this does not change the scientific conclusions of the article in any way. The original article has been updated.

## Publisher’s Note

All claims expressed in this article are solely those of the authors and do not necessarily represent those of their affiliated organizations, or those of the publisher, the editors and the reviewers. Any product that may be evaluated in this article, or claim that may be made by its manufacturer, is not guaranteed or endorsed by the publisher.

