# Supplementary material for: Combinatorial Strategies to Target Molecular and Signaling Pathways to Disarm Cancer Stem Cells
Source: Front Oncol. 2021 Jul 26;11:689131. doi: 10.3389/fonc.2021.689131 (PMC8352560; doi:10.3389/fonc.2021.689131)
Supplement: Supplementary file 1 [file Table_1.docx]

| **Tumor** | **Combinatorial therapy** | **Molecular**  **target** | **Phenotipic**  **outcome** | **Cellular/animal models** | **References** |
| --- | --- | --- | --- | --- | --- |
| Medulloblastoma | Alpelisib +  CGP57380 | PI3Kα + MNK | Impairment of neurosphere growth  Reduction of tumor growth, increase of mice survival | Medulloblastoma CSCs  Xenograft animal model | [90] |
| Neuroblastoma | CCT128930 + PD98059 | AKT2 + MEK | Reduction of CSCs  sphere formation, cell proliferation, and cell migration | Neuroblastoma CSCs | [91] |
| Prostate cancer | Napabucasin + docetaxel | STAT3 | Inhibition of CSCs cell proliferation | Prostate CSCs | [110] |
| Prostate cancer | BEZ235 + radiation | PI3K + mTOR | Reduction of EMT, CSCs markers and self-renewal proteins expression, CSCs colony formation ability;  induction of apoptosis | Prostate CSCs | [111] |
| Non-small cell lung cancer | Napabucasin + cisplatin | STAT3 | Reduction of CSCs markers, impairment of CSCs proliferation and clonogenic survival, induction of CSCs apoptosis | Non-small-cell lung cancer CSCs | [113] |
| Head and neck squamous cell carcinoma | SB203580 + cisplatin | p38 | Decrease of survival and colony forming ability, increase of apoptosis, impairment of DNA repair capacity and CSCs maintenance | Head and neck squamous cell carcinoma CSCs | [114] |
| Head and neck squamous cell carcinoma | PTC-209 + cisplatin | BMI1 | Reduction of the BMI1^+^  CSCs-mediated lymph node metastases | C57BL/6, NOD/SCID and nude mice bearing 4NQO-induced  Head and neck squamous cell carcinoma | [116] |
| Head and neck squamous cell carcinoma | Dasatinib +  EC-8042 | Src | Reduction of HNSCC cells  invasion ability,  Reduction of viability of CSCs-enriched tumorspheres and the expression of CSCs-related factors  Reduction of tumor growth, increase in mice survival, reduction of Ki67-positive cells, increase of apoptotic cells, decrease in the CSCs markers expression, reduction of tumorspheres-formation | Head and neck squamous cell carcinoma CSCs  Head and neck squamous cell carcinoma xenograft animal model | [117] |
| Colorectal cancer | LGK-794 +5-FU | WNT | Suppression of tumor growth | PDX mouse model | [122] |

**Supplementary Table1. Combinatorial therapies assessed in various types of cancers.**

| Colon cancer | Dasatinib + curcumin | Src | Inhibition of CSCs growth, colonosphere formation, invasion potential and markers  Reduction of CSCs markers and CSCs population | Colon CSCs  APCMin+/- mice | [123] |
| --- | --- | --- | --- | --- | --- |
| Hepatocellular carcinoma | RO3306 + sorafenib | CDK1 + Tyrosine kinase PDK1 | Suppression of CSCs proliferation and tumor growth | PDX animal model | [124] |
| High-grade serous ovarian cancer | Saracatinib + selumetinib | Src + MEK1/2 | Reduction of CSCs population and spheres formation  Impairment of tumor growth | Ovarian CSCs  Xenograft animal model | [33] |
| Pancreatic cancer | Dasatinib + gemcitabine | Src | Inhibition of CSCs proliferation and survival, induction of apoptosis | Pancreatic CSCs | [125] |
| Pancreatic cancer | SB431542 + gemcitabine  SB431542 + gemcitabine + CUR199691 | Nodal  Nodal + Shh | S phase arrest and apoptosis induction  Delay of tumor growth and increase of mice survival  Impairment of tumor growth and increase of mice survival | Pancreatic CSCs  Pancreatic cancer xenograft mouse model  PDX animal model | [105] |
| Melanoma | anti-Nodal antibody + dacarbazine (DTIC) | Nodal | Decrease in cell vitality, suppression of proliferation, induction of apoptosis | Melanoma CSCs  Multicellular tumor spheroid culture | [107] |
| Melanoma | anti-Nodal antibody + RG7204 (vemurafenib) | Nodal + B-RAF | Decrease in viability, increase in cell death | Melanoma cell line harboring the active V600E B-RAF | [107] |
| Melanoma | anti-Nodal antibody + dabrafenib inhibitor of BRAF | Nodal + B-RAF | Reduction of anchorage-independent colony formation and tumorigenic growth potential | V600E B-RAF metastatic melanoma cell line | [126] |
| Esophageal cancer | SNX-2112 +  STAT3 Sh-mediated knockdown | Hsp90 + STAT3 | Inhibition of esophageal cancer stem-like cells (ECSLCs) proliferation, ECSLCs G2/M phase arrest,  induction of ECSLCs apoptosis    Reduction of colony formation,  and tumor growth | Esophageal cancer stem-like cells (ECSLCs)  ECSLCs xenograft animal model | [132] |
